# A Fast and Simple Solid Phase Extraction-Based Method for Glucosinolate Determination: An Alternative to the ISO-9167 Method

**DOI:** 10.3390/foods13050650

**Published:** 2024-02-21

**Authors:** Yanfang Li, Mengliang Zhang, Pamela Pehrsson, James M. Harnly, Pei Chen, Jianghao Sun

**Affiliations:** 1 Methods and Application of Food Composition Laboratory, Beltsville Human Nutrition Research Center, Agricultural Research Service, U.S. Department of Agriculture, Beltsville, MD 20705, USA; yanfang.li@usda.gov (Y.L.); pamela.pehrsson@usda.gov (P.P.); james.harnly@usda.gov (J.M.H.); pei.chen@usda.gov (P.C.); 2 Department of Chemistry, Middle Tennessee State University, Murfreesboro, TN 37132, USA; mengliang.zhang@mtsu.edu

**Keywords:** glucosinolates, quantification, ISO 9167 method, *Brassicaceae* vegetables, dimethylaminopropyl (DEA), weak anion exchange, solid phase extraction (SPE) cartridge

## Abstract

Glucosinolates (GLSs) are a well-studied sulfur-containing compound found in *Brassicaceae* plants that play critical roles in plant resistance and human health. Correctly identifying and reliably quantifying the total and individual GLS content is of great importance. An improved method as an alternative to the ISO 9167-1 (ISO) method is developed in the present study. An efficient extraction and purification procedure is proposed with a commercially available dimethylaminopropyl (DEA)-based weak anion exchange solid-phase extraction (SPE) cartridge instead of using the self-prepared ion-exchange columns in the ISO method. The GLSs are identified and quantified by ultra high-performance liquid chromatography (UHPLC) high-resolution mass spectrometry (HRMS). The method demonstrates a comparable quantification of total and individual GLSs on certified rapeseeds and other *Brassicaceae* vegetables when compared to the ISO method. The developed SPE method is simpler and more efficient, thus allowing for applications to a large sample size with reduced analysis time, improved repeatability and accuracy, and possible automation.

## 1. Introduction

Glucosinolates (GLSs) are a group of highly investigated, sulfur-containing secondary metabolites that are abundant in the *Brassicaceae* family, such as broccoli, mustard, kale, cabbage, etc. [1]. GLSs are water-soluble *N*-hydroxy sulfates that share a basic structure of a thioglucose moiety, a sulfonated oxime moiety, and a variable side chain derived from amino acids, and it is normally named the R-group [2]. There have been about 137fully or partially characterized GLSs reported, and they differ from each other by the R group [3,4]. According to the structure of the R-group, GLSs are classified as aliphatic, indole, or aromatic [5]. GLSs and their metabolites have been observed to have potential health-beneficial properties in epidemiological studies, thereby indicating protective effects against various cancers, such as breast [6], prostate [7], colorectal [8], stomach [9], bladder [10], renal [11], and lung cancers [12]. The consumption of GLSs containing *Brassicaceae* vegetables is also associated with lower risks of chronic diseases, particularly neurodegenerative diseases, cardiovascular diseases [13], and obesity [1].

The accurate quantification of GLSs in plant materials is crucial for nutritional labeling, dietary assessment, and plant breeding programs, which can significantly impact the development of food products, clinical studies, and crops. The inaccurate quantification of GLSs can lead to misleading results in human nutritional studies and the mislabeling of commercial products. Quantifying the total or individual GLSs, however, has faced challenges due to a lack of pure GLS reference compounds [14]. Techniques such as HPLC and LC-MS have been widely employed for the detection and quantification of GLSs [15,16]. With limited commercially available reference standards, it is very challenging to detect and quantify GLSs using conventional LC-MS. Furthermore, GLSs are highly polar due to the presence of thioglucosyl and sulfonated oxime moieties, thus making them chromatographically unfavorable in a reversed-phase HPLC system; therefore, the sufficient separation of individual intact GLSs is difficult [17,18]. Consequently, a widely accepted method for the quantification of total and individual GLSs involves desulfation, which is used to remove the sulfate group before using HPLC with diode array detection (DAD), and this is achieved by relying on the relative response factors (RRFs) of desulfo-GLSs established by the European Community (ISO 9167:2019, Rapeseed and Rapeseed Meals—Determination of Glucosinolates Content—Method Using High-Performance Liquid Chromatography. International Organization for Standardization. Geneva, Switzerland, 2019. https://www.iso.org/standard/72207.html, accessed on 16 February 2024).

The ISO 9167-1:2019 (ISO) method extracts and purifies GLSs using self-prepared ion-exchange columns containing DEAE Sepharose CL-6B (or Sephadex A25) resin as sorbents. Although the ISO method has been widely adopted in the scientific community for the purposes of studying different food or botanical samples [19,20], there is a notable limitation as commercial Sepharose CL-6B (or Sephadex A25) anion exchange columns are not readily available. The preparation of columns with DEAE Sepharose CL-6B (or Sephadex A25) resin is time-consuming, exhibits low repeatability, is less user-friendly for new operators, and proves challenging to scale up for a large number of samples [21,22]. A more efficient and precise extraction and quantification method that improves upon the dated ISO method is needed.

GLSs exist as anions in nature; as such, one promising approach to address these challenges is solid-phase extraction (SPE) with an anion exchange cartridge, which would simplify the ISO method and improve its accuracy, precision, and efficiency. SPE is widely used for sample cleanup in general food and environmental analysis [23]. These cartridges can selectively retain GLSs and facilitate their purification from complex plant matrices, thus potentially decreasing interferences and enhancing the extraction repeatability and quantification precision.

This study aimed to develop a new method with commercially available solid-phase extraction (SPE) cartridges for the quantification of total and individual GLSs as an alternative to the ISO method. The feasibility of five commercial anion exchange SPE cartridges was tested for the extraction and purification of GLSs, where the aim was to simplify the complexity of the ISO method and to contribute to easier and faster operation, as well as to deliver greater method precision for botanical materials. The elution procedures, buffer, and hydrolysis steps were also optimized for the selected SPE cartridges. Additionally, four varieties of mustard seeds and six different vegetables were comparatively investigated with the SPE and the ISO methods.

## 2. Materials and Methods

### 2.1. Chemicals

Sinigrin potassium salt (≥95.0%), imidazole formate (≥94%), DEAE Sepharose CL-6B suspension and sulfatase (from *Helix pomatia*, Type H-2, aqueous solution, ≥2000 units/mL), and certified rapeseeds (ERM-BC367) were purchased from Sigma-Aldrich (St. Louis, MO, USA). The sulfatase was diluted 100-fold with ultrapure water to a working solution. Formic acid and liquid chromatography-mass spectrometry (LC-MS) grade acetonitrile were purchased from Fisher Scientific (Waltham, MA, USA). Ultrapure water was prepared from distilled water using a Milli-Q system (Millipore Lab., Bedford, MA, USA). All other chemicals and reagents were of analytical grade.

### 2.2. Sample Information

Four varieties of mustard seeds were obtained from a local market. Seed samples were ground into fine powder and then passed through a 40-mesh sieve. The powder was dried using a Labconco Freezone lyophilizer (Kansas City, MO, USA). Six microgreen Brassicaceae vegetables, including red cabbage, broccoli, arugula, kale, China rose radish, and collard were obtained from the Food Quality Laboratory of USDA-ARS. All fresh samples were lyophilized and then powdered with an analytical mill (Ika A11 Basic, Wilmington, NC, USA).

### 2.3. Glucosinolates Extraction

Two hundred milligrams of sample were transferred to a 15 mL pre-heated tube (75 °C, 1 min), which was followed by adding 5 mL of 70% methanol. The liquid level on the tube was marked after vortexing for 30 s. Then, 200 µL of an internal standard (IS) solution (5 mmol/L sinigrin monohydrate) was added to the tube. The tube was placed in a 75 °C water bath for 20 min. Additional methanol was added to return the liquid level in the tube back to the mark after cooling to room temperature. The supernatant was recovered after centrifugation for 15 min at 5000 rpm. The supernatant was filtered with a 0.45 μm PVDF syringe filter and stored at −20 °C until analyzed.

### 2.4. SPE Cartridges

Oasis WAX 3cc (60 mg) flangeless cartridge and Oasis MAX 3cc (60 mg) extraction cartridges were purchased from Waters Corp. (Milford, MA, USA). A Strata X-AW 33 µm polymeric weak anion (30 mg/1 mL) cartridge was purchased from Phenomenex (Torrance, CA, USA). Bond Elut DEA (100 mg, 1 mL) and Bond Elut NH_2_ (100 mg, 1 mL) SPE cartridges were purchased from Agilent Technologies (Palo Alto, CA, USA).

### 2.5. Purification of Glucosinolates

First, the SPE cartridges were inserted into the ports of an SPE Vacuum Manifold, which was connected to a vacuum source and waste trap. Different SPE cartridges were conditioned with instructions provided by the manufacturers with little modifications, as shown in Figure S1.

The Oasis MAX 3cc (60 mg) extraction cartridge (Figure S1A) was conditioned and equilibrated with two doses of 1 mL of methanol and two doses of 1 mL of water, respectively. After loading 1 mL of sample, the cartridge was washed with a 2 × 1 mL 5% NH_4_OH aqueous solution (*v/v*) and 2 × 1 mL methanol, respectively. Finally, the analytes were eluted with 2 × 1 mL of 2% FA methanol solution (*v/v*). All the elutes from the sample loading step were collected and analyzed by UHPLC-HRMS (Figure S1A). For instance, the solutions that were collected from sample loading, twice washing, and elution steps were labeled as A1, A2, A3, and A4, respectively. UHPLC-HRMS was used to monitor the elution of the GLSs in each step to evaluate the effectiveness of GLS extraction. Similar procedures were repeated for the other tested SPE cartridges.

The Bond Elut NH_2_ SPE cartridge (Figure S1B) was conditioned and equilibrated with two doses of 1 mL of methanol and two doses of 1 mL of a 1% acetic acid (AA) aqueous solution (*v/v*). After loading 1 mL of sample, the SPE cartridge was washed twice with 1 mL of a 5% AA methanol solution (*v/v*). Finally, the analytes were eluted with 2 × 1 mL of a 5% NH_4_OH methanol solution. The eluted solutions were labeled as B1, B2, and B3 after the loading, washing, and elution steps, respectively.

The Strata X-AW polymeric weak anion SPE cartridge (Figure S1C) was conditioned and equilibrated with 2 × 1 mL of methanol and 2 × 1 mL of water, respectively. After loading 1 mL of sample, the column was washed with 2 × 1 mL of an ammonium acetate aqueous solution (*v/v*) and 2 × 1 mL of methanol, respectively. Finally, the analytes were eluted with 2 × 1 mL of a 5% NH_4_OH methanol solution. The eluted solutions were labeled as C1, C2, C3, and C4 after the loading, twice washing, and elution steps, respectively.

The Oasis WAX 3cc (60 mg) flangeless cartridge (Figure S1D) was conditioned and equilibrated with two doses of 1 mL of methanol and two doses of 1 mL of water, respectively. A 1 mL sample was mixed with a 1 mL of a 2% FA aqueous solution (*v/v*), which was next loaded into the SPE cartridge. Then, the cartridge was washed with 2 × 1 mL of a 2% FA aqueous solution (*v/v*) and 2 × 1 mL of methanol, respectively. Finally, the analytes were eluted with 2 × 1 mL of a 5% NH_4_OH methanol solution. The eluted solutions were labeled as D1, D2, D3, and D4 after the loading, twice washing, and elution steps, respectively.

The Bond Elut DEA SPE cartridge (Figure S1E) was conditioned and equilibrated with two doses of 1 mL of methanol and two doses of 1 mL of 1% formic acid (FA) aqueous solution (*v/v*). After loading 1 mL of sample, the SPE cartridge was washed twice with 1 mL of a 2% FA aqueous solution (*v/v*). Finally, the analytes were eluted with 2 × 1 mL of a 5% NH_4_OH aqueous solution. The eluted solutions were labeled as E1, E2, and E3 after the loading, washing, and elution steps, respectively.

### 2.6. Desulfation of Glucosinolates

**Figure 1:**
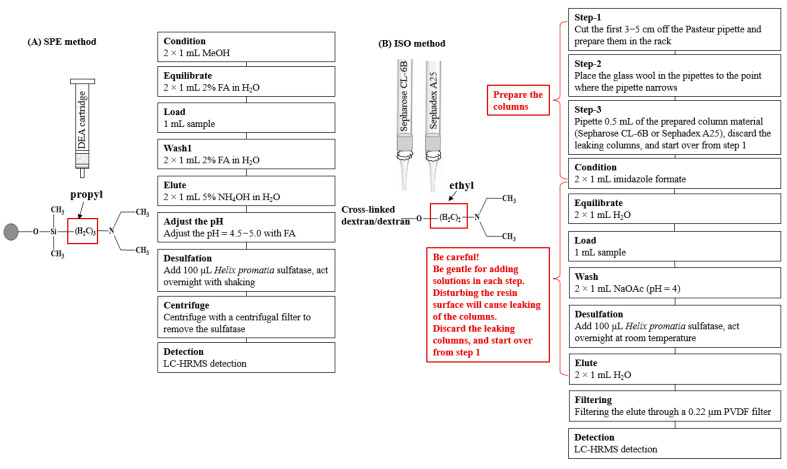
Scheme of (**A**) the SPE method and (**B**) the ISO method. FA, formic acid; MeOH, methanol; NH_4_OH, ammonium hydroxide; and NaOAc, sodium acetate.

The Bond Elut DEA SPE column was selected (see the Results and Discussion section) for the study of desulfation (Figure 1). After eluting the DEA SPE cartridge with 2 × 1 mL of 5% NH_4_OH, the pH of the eluent was adjusted with formic acid to a pH = 4.5−5.0 using a pH strip. Then, 100 μL of a sulfatase working solution was added to the acidic solution to react overnight at room temperature with shaking. The desulfo-GLSs were separated from the sulfatase by centrifuging them with a centrifugal filter (3 k Dalton molecular weight cutoff) for 15 min at 10,000 rpm. The desulfo-GLSs were collected and stored at −20 °C for further analysis. Each sample was prepared in triplicate.

### 2.7. Purification and Desulfation of Glucosinolates with the ISO Method

A second set of samples were prepared following the ISO method with slight modifications (Figure 1). Firstly, the ion exchange columns were prepared by cutting the first 3–5 cm off the Pasteur pipette and putting them in the rack, placing the glass wool in the pipettes to the point where the pipette narrows, and then by pipetting 0.5 mL of the prepared ion-exchange resin (Sepharose CL-6B). After draining off the water, wash with two aliquots of 1 mL imidazole formate (6 mol/L) solutions and two aliquots of 1 mL of H_2_O. The column was loaded with 1 mL of sample and washed twice with 1 mL of sodium acetate. The column was allowed to drain after each addition. It should be noted that particular care should be taken with each step when adding solutions to the homemade columns to avoid disturbing the resin surface, which will cause a leaking of the columns. Should leaking occur, discard the column and start over from the beginning. Finally, 100 µL of the sulfatase working solution was added, which was then left to react overnight at ambient temperature. The desulfo-GLSs were eluted by adding two aliquots of 1 mL of water. The elute was mixed and filtered through a 0.22 µm PVDF filter before injecting for UHPLC-HRMS analysis.

### 2.8. UHPLC-HRMS Conditions for the Detection of Glucosinolates

The collected elutes were analyzed by UHPLC-HRMS (a Vanquish UHPLC coupled with an Orbitrap Fusion ID-X Tribrid mass spectrometer) with an Agilent Eclipse Plus-C_18_ column (150 mm × 2.1 mm i.d., 1.8 μm) (Santa Clara, CA, USA), which was combined with an UltraShield pre-column. The flow rate was 0.3 mL/min. The injection volume was 2 μL. The column temperature was set as 60 °C. Mobile phase A was 0.1% formic acid in water (*v/v*), and mobile phase B was 0.1% formic acid in acetonitrile (*v/v*). The pre-equilibration was set as 2% mobile phase B for 5 min. The linear gradient was from 2 to 35% B at 20 min, to 95% B at 25 min. The UV signal was monitored at 229 nm. The mass was scanned for 25 min in negative mode with electrospray ionization (ESI). The negative ion voltage was 2500 V. The sheath, aux, and sweep gas were set as 40, 10, and 5 Arb, respectively. The ion transfer tube and vaporizer temperatures were 300 and 275 °C, respectively. The Orbitrap resolution was 60,000 and the scan range (*m*/*z*) was 120–2000.

### 2.9. Quantification of the Total and Individual Glucosinolates

The quantification of total and individual GLSs was based on the ISO method. The calculation was based on the equation $\frac{Ag}{As}\times \frac{n}{m}\times Kg\times \frac{100}{100-w}$, where *A_g_* and *A_s_* are the peak area of the corresponding desulfo-GLS and desulfo-sinigrin (internal standard), respectively; *n* is the micromoles of the internal standard (*n* = 200 µL × 5 mmol/L = 1 µmol); *m* is the weight of the test sample; *K_g_* is the relative response factor of the desulfo-GLSs that are provided in the ISO method (average *K_g_* value was used for the desulfo-GLSs that not fully separated); and w is the percentage of moisture and volatile matter of the test sample (*w* = 5.7 for the certified rapeseeds ERM-BC367 as described in the manufacture report, *w* = 0 for the tested *Brassicaceae* vegetables for dry materials that were used). The final contents of the individual and total GLSs were expressed as µmol/g dry material.

### 2.10. Statistical Analysis

Data were expressed as the mean value ± standard deviation (SD) (*n* = 3). Paired t-tests were used to analyze the statistical differences between groups and were performed using SPSS Statistics (version 25.0, SPSS, Inc., Chicago, IL, USA), with *p* < 0.05 considered statistically significant. Figures were prepared using GraphPad Prism (version 8.0, Graphpad Software Inc., San Diego, CA, USA).

## 3. Results and Discussion

### 3.1. Selection of Different Commercial Ion-Exchange SPE Cartridges

In this study, certified rapeseed ERM-BC367 was used to evaluate the extraction efficiency of the GLSs with five commercial SPE cartridges from different manufacturers. The optimization of an appropriate SPE cartridge with proper sorbent materials plays a key role in the achievement of reproducible recovery for GLSs. Ion exchange SPE cartridges can separate charged analytes in a solution based on electrostatic interactions between the analytes and the charged sorbents. Strong ion exchanges are used for weak acidic/basic substances, while weak ion exchanges are used for strong acidic/basic analytes [24]. Since GLSs are anionic compounds, SPE cartridges with weak anion exchange or mixed-mode sorbent, which remain charged and were maintained at a consistently high capacity in acidic conditions, were selected in this study. To test whether the selected SPE cartridges were appropriate for extraction of GLSs, the eluted solutions from each step after loading the samples were analyzed with UHPLC-HRMS to evaluate the presence of the intact GLSs. The working procedures for the selected SPE cartridges are shown in Figure S1.

For Oasis MAX SPE cartridge (A), intact GLSs were not detected in all of the steps after sample loading (A1–A4 in Figure S1 and Table 1), thus indicating that the GLSs were attached on this SPE cartridge but were not successfully eluted. The Oasis MAX cartridges contain mixed-mode polymeric sorbent with anion-exchange groups, and they are designed for extracting acidic compounds. However, due to the strong ion-exchange interactions between the stationary phase and polar metabolites, the elution requires high salt concentrations in the elution solvent [25], which was not used in the current study. This may result in no GLSs in the eluting step. Since the presence of salt may decrease the accuracy for mass measurement, the elution condition was not modified, and the Oasis MAX SPE cartridge was not used in the later stages of the study. With Bond Elut NH_2_ (B) and Strata-X-AW SPE (C) cartridges, the intact GLSs were not detected after loading samples (B1 and C1 in Figure S1 and Table 1, respectively); however, they were detected in the washing steps (B2 and C2–C3 in Figure S1 and Table 1, respectively), thus indicating the washing conditions were not proper for cartridges B and C. In addition, with the Oasis WAX SPE (D) and Bond Elut DEA (E) cartridges, the GLSs were detected only in the eluting steps (D4 and E3 in Figure S1 and Table 1, respectively), but not in the sample loading and washing steps (D1–D3 and E1–E2 in Figure S1 and Table 1, respectively), thus suggesting that these two cartridges could be used for GLS extraction. However, the Oasis WAX SPE cartridge (D) was environmentally unfriendly because more organic solvent was needed in the washing and eluting steps. Therefore, the Bond Elut DEA SPE cartridge (E) was used for further study.

### 3.2. Method Optimization

Quantification of the individual GLSs with the ISO method relies on the RRFs of desulfo-GLS. To use RRFs for the quantification of the individual GLS in the present study, intact GLSs were also enzymatically desulfated. To achieve working conditions for the sulfatase, the pH of the elute from DEA SPE cartridge was adjusted to 4.5–5.0 before adding the working sulfatase solution. The complete strategy for GLS extraction was developed, as shown in Figure 1. To comparatively investigate the SPE method and the ISO method, ERM-BC367 was used as a reference sample and tested in triplicate using 200 mg following the protocol described in the experimental section (Figure 1). Due to cleavage of the sulfur–aglycone bond, the thioglucosyl fragment ion (*m/z* 195) was formed, which was used as a characteristic ion for desulfo-GLSs in the MS data [26]. Therefore, the extracted ion chromatograms (EIC) of *m/z* 195 were presented, as shown in Figure S2, to demonstrate the desulfo-GLSs in ERM-BC367 following the ISO and SPE methods. The desulfo-GLSs were confirmed by retention time, accurate molecular mass, the fragment ion mass obtained via UHPLC-HRMS and online databases (e.g., SciFinder, PubChem), and the published literature.

**Figure 2:**
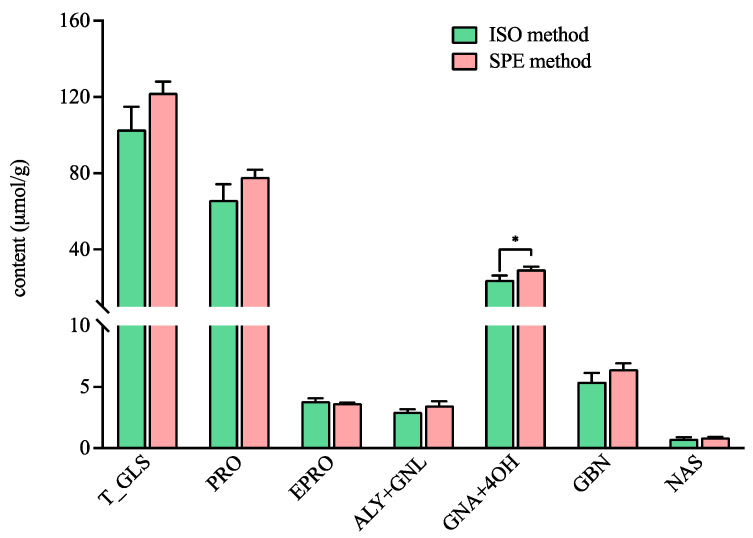
The contents of the total and individual glucosinolates in the certified rapeseed ERM-BC367 reference using the ISO and SPE methods. Total glucosinolates (T_GLS); progoitrin (PRO); epiprogoitrin (EPRO); glucoalyssin (ALY); gluconapoleiferin (GNL); gluconapin (GNA); 4-hydroxyglucobrassicin (4OH); glucobrassicanapin (GBN); and gluconasturtiin (NAS). * significantly different (*p* < 0.05) as determined via a paired *t*-test.

The total content of GLSs detected by the ISO method in ERM-BC367 was in accordance with the value in the manufacture report (103.05 ± 11.79 µmol/g vs. 99 ± 9 µmol/g) [27]. In addition, there was no significant difference for the total GLS content in ERM-BC367 detected by the ISO method and the SPE method (Figure 2). The content of the individual GLS, including progoitrin (PRO), epiprogoitrin (EPRO), glucoalyssin (ALY) and gluconapoleiferin (GNL), glucobrassicanapin (GBN), and gluconasturtiin (NAS), were comparable between both methods (Figure 2), thus indicating that the SPE method can serve as an alternative to the ISO method. The functional group for the DEAE Sepharose CL-6B resin and DEA SPE cartridge are both tertiary amine (-N(CH_3_)_2_), which can interact with the anionic sulfate part (-SO_3_-) of GLSs, thereby leading to the comparable adsorption capacity of the GLSs. Interestingly, the content of the total gluconapin (GNA) and 4-hydroxyglucobrassicin (4OH) detected by the SPE method was significantly higher than that detected by the ISO method (Figure 2). The ligands connected with the functional group were a propyl group (-CH_2_-)_3_ for the DEA SPE cartridge and an ethyl group (-CH_2_-)_2_ for the DEAE Sepharose CL-6B resin. The occurrence of an additional methylene group (-CH_2_-) may cause the difference in the absorbance of individual GLSs. It has been reported that more methyl groups in the resin’s functional group may lead to higher absorption capacity for GLSs [28]. However, the effect of the carbon number in the ligand has not been reported yet; thus, it needs further investigation. Furthermore, smaller error bars were observed in the content of the total GLSs and in the contents of the PRO, EPRO, GNA + 4OH, GBN, and NAS detected by the SPE method than that detected by the ISO method, thereby suggesting better repeatability with the SPE method.

### 3.3. Application in Brassicaceae Vegetables Samples

The applicability of this new method was comparatively investigated with the ISO method for the quantification of total and individual GLSs in four varieties of mustard seeds and six leafy greens (red cabbage, broccoli, arugula, kale, China rose radish, and collard). The GLSs were identified by HRMS data and retention times and were compared to the certified rapeseed reference material (ERM-BC367), which was used as an authentic reference in this study. The GLSs not certified in ERM-BC367 were tentatively identified based on HRMS data and the results in the published literature. The concentrations of the total and individual GLSs were calculated based on the peak areas of the UV response at 229 nm using the equation described in the method section, with sinigrin as the internal standard (IS).

**Figure 3:**
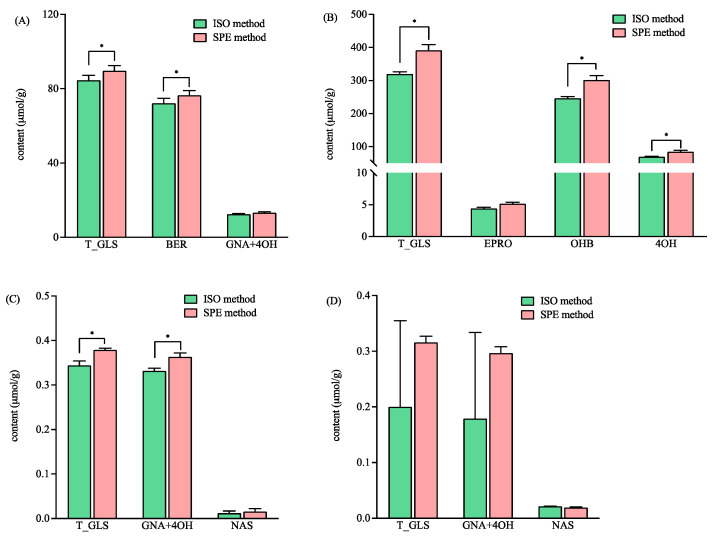
The contents of total and individual glucosinolates in four different mustard seeds using the ISO and SPE methods. (**A**–**D**) represent the mustards of YL03, WT04, BN01 and Hua01, respectively. Total glucosinolates (T_GLS); glucoiberin (BER); gluconapin (GNA); 4-hydroxyglucobrassicin (4OH); epiprogoitrin (EPRO); hydroxybenzyl glucosinolate (OHB); and gluconasturtiin (NAS). * significantly different (*p* < 0.05) as determined via a paired *t*-test.

As shown in Figure 3, the total GLS concentrations ranged broadly in the four varieties of mustard seeds from 0.2 to 319.70 and 0.32 to 391.13 µmol/g, as detected by the ISO and SPE methods, respectively. The concentrations of the total GLSs in variety WT04 was 100 times higher than that in variety Hua01 when detected by both methods (Figure 3). The total contents of GLSs detected by the SPE method was significantly higher than that detected by the ISO method in three varieties of mustard seeds, namely YL03, WT04, and BN01 (Figure 3). Furthermore, the HRMS data showed that intact BER was detected in the sample of YL03 treated by the ISO and the SPE methods. The EIC intensities for intact BER (*m*/*z* 422) detected by the SPE method were significantly higher than that detected by the ISO method (*p* < 0.05) (3.64 ± 0.47 (×10^8^) vs. 1.49 ± 0.46 (×10^8^)). The results indicated that more BER was retained in the commercial DEA SPE cartridge than in the self-prepared Sepharose CL-6B column that was used in the ISO method.

For the variety WT04, the contents of hydroxybenzyl glucosinolate (OHB) and 4-hydroxyglucobrassicin (4OH) detected by the SPE method were significantly higher than that detected by the ISO method (Figure 3B). In the variety BN01, gluconapin (GNA) and 4-hydroxyglucobrassicin (4OH) were not fully separated in the chromatogram, and their content were calculated combined. The total content of the GNA and 4OH detected by the SPE method was significantly higher than that detected by the ISO method (Figure 3C). In Hua01, better precision was obtained with the SPE method for the total GLSs, GNA, and 4OH (Figure 3D). In addition, the GLS profiles were found to be variety-dependent (Figure 3). This agreed with the previous report that mustard seeds show different glucosinolate profiles based on their varieties [29].

**Figure 4:**
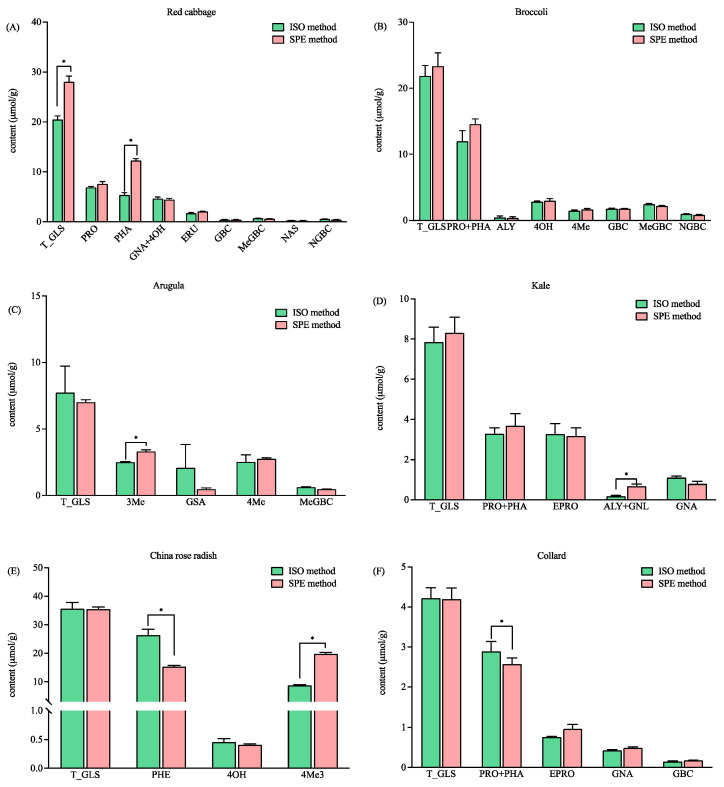
The contents of the total and individual glucosinolates in six vegetables using the ISO and SPE methods. (**A**) Red cabbage; (**B**) broccoli; (**C**) arugula; (**D**) kale; (**E**) China rose radish; and (**F**) collard. Total glucosinolates (T_GLS); progoitrin (PRO); glucoraphanin (PHA); gluconapin (GNA); 4-hydroxyglucobrassicin (4OH); glucoerucin (ERU); glucobrassicin (GBC); methoxyglucobrassicin (MeGBC); gluconasturtiin (NAS); neoglucobrassicin (NGBC); glucoalyssin (ALY); 4-Methylthiobutyl glucosinolate (4Me); 3-Methylsulfinylbutyl glucosinolate (3Me); glucosativin (GSA); gluconapoleiferin (GNL); glucoraphenin (PHE); 4-methylthio-3-butenylglucosinolate (4Me3); and epiprogoitrin (EPRO). * significantly different (*p* < 0.05) as determined via a paired t-test.

For the six *Brassicaceae* vegetables studied, the total concentration of the GLSs in red cabbage detected by the SPE method was 26.61 µmol/g, which is significantly higher than that detected by the ISO method (i.e., 18.85 µmol/g (Figure 4A)). The content of glucoraphanin (PHA) in red cabbage was found to be significantly higher with the SPE method (Figure 4A). It is worth noting that the intact PHA in red cabbage was detected with both methods, and they were also confirmed by the HRMS data. The EIC revealed that the intensities of total intact (*m*/*z* 436) and desulfo-PHA (*m*/*z* 356) were the same for the SPE and ISO methods (*p* > 0.05). The results indicated that comparable levels of PHA were retained in the DEA cartridge and the self-made column. However, more PHA were desulfated and eluted in the SPE method (Figure 4A). The results, as shown in Figure 3A and Figure 4A, suggested that both methods may not ensure the full desulfation of all GLSs, which can lead to the incorrect quantification of GLSs. Therefore, it is necessary to involve HRMS to validate the analysis. The total and individual GLSs in the broccoli detected with both methods were comparable (Figure 4B). The total GLS content detected in this study (21.86 µmol/g vs. 23.33 µmol/g, *p* > 0.05) was higher than that reported in 53 different broccoli samples (9.91 µmol/g) and 15 Tenderstem^®^ broccoli samples (19.53 µmol/g) [19], thus indicating that GLS content varies according to different varieties. The content of 3-methylsulfinylbutyl glucosinolate (3Me) in arugula, the total content of glucoalyssin and gluconapoleiferin (ALY + GNL) in kale, and the content of 4-methylthio-3-butenylglucosinolate (4Me3) in China rose radish were found to be significantly higher with the SPE method (Figure 4C–E). In addition, the content of glucoraphenin (PHE) in China rose radish and the total content of progoitrin and glucoraphanin (PRO + PHA) in collard detected by the ISO method were significantly higher than those detected by the SPE method. However, the total GLS content when detected by the two methods (Figure 4E,F) was comparable. The results suggested that the method that was developed based on anion exchange solid phase extraction has better, or at least comparable, ability for the detection of total GLS levels than the ISO method. In addition, it has the advantages of labor saving and better repeatability. The extraction ability of the two methods for the individual GLSs were different, which may be caused by the different number of methylene groups in the ligand. However, more investigation is needed.

## 4. Conclusions

The present study developed an anion exchange SPE-based method as an alternative to the whole ISO method for a faster, easier, and more reproducible quantification of the total and individual GLSs in *Brassicaceae* vegetables. Five commercially available SPE cartridges were investigated for their ability to retain the GLSs from vegetable extracts. A Bond Elut DEA SPE cartridge was selected as it has the best ability to retain GLSs. The elution procedures, buffer, and hydrolysis steps were optimized to produce desulfo-GLSs. Higher or comparable contents of the total or individual GLSs in ERM-BC367 were detected by the SPE method. The applicability of the SPE method was illustrated by the identification and quantification of GLSs in ten *Brassicaceae* vegetables. Though the concentrations of some individual GLSs were found to be different with these two detection methods, comparable contents of the total GLSs were detected with both methods. The SPE-based method can be used as an alternative to the ISO method for the detection of total GLSs with higher throughput and a more automated fashion.

## Figures and Tables

**Table 1:** Occurrence of glucosinolates in the collected solutions of each step in different commercial SPE cartridges (Figure S1), as detected by UHPLC-HRMS.

Cartridges	Oasis MAX (A)		Bond Elut NH_2_ (B)		Strata-X-AW (C)		Oasis WAX (D)		Bond Elut DEA (E)	
Sample load	A1		B1		C1		D1		E1	
Wash 1	A2		B2	√	C2	√	D2		E2	
Wash 2	A3				C3	√	D3			
Elution	A4		B3	√	C4	√	D4	√	E3	√

√ means intact glucosinolates were detected.

## Data Availability

The original contributions presented in the study are included in the article/supplementary material; further inquiries can be directed to the corresponding author.

## Supplementary Materials

The following supporting information can be downloaded at: https://www.mdpi.com/article/10.3390/foods13050650/s1, Figure S1: Scheme of the purification of glucosinolates extracted from certified rapeseeds (ERM-BC367) with different commercial SPE cartridges: (A) Oasis MAX; (B) Bond Elut NH_2_; (C) Strata-X-AW; (D) Oasis WAX; (E) and Bond Elut DEA; Figure S2. Extracted ion chromatogram (EIC) (*m*/*z* 195) of the certified rapeseed reference (ERM-BC367) following (A) the ISO method and (B) SPE method.
