# Supplementary material for: A Fast and Simple Solid Phase Extraction-Based Method for Glucosinolate Determination: An Alternative to the ISO-9167 Method
Source: Foods. 2024 Feb 21;13(5):650. doi: 10.3390/foods13050650 (PMC10931338; doi:10.3390/foods13050650)
Supplement: Supplementary file 1 [file foods-13-00650-s001.zip › foods-2857406-supplementary.pdf]

Supplemental information for

A Fast and Simple Solid Phase Extraction-Based Method for Glucosinolate Determination: An Alternative to the ISO-9167 Method

Yanfang Li [1,2], Mengliang Zhang [2], Pamela Pehrsson [1], James M. Harnly [1], Pei Chen [1] and Jianghao Sun [1,*]

[1] Methods and Application of Food Composition Laboratory, Beltsville Human Nutrition Research Center, Agricultural Research Service, U.S. Department of Agriculture, Beltsville, MD 20705, USA; yanfang.li@usda.gov (Y.L.); pamela.pehrsson@usda.gov (P.P.); james.harnly@usda.gov (J.M.H.); pei.chen@usda.gov (P.C.)

[2] Department of Chemistry, Middle Tennessee State University, Murfreesboro, TN 37132, USA; mengliang.zhang@mtsu.edu

* Correspondence: jianghao.sun@usda.gov

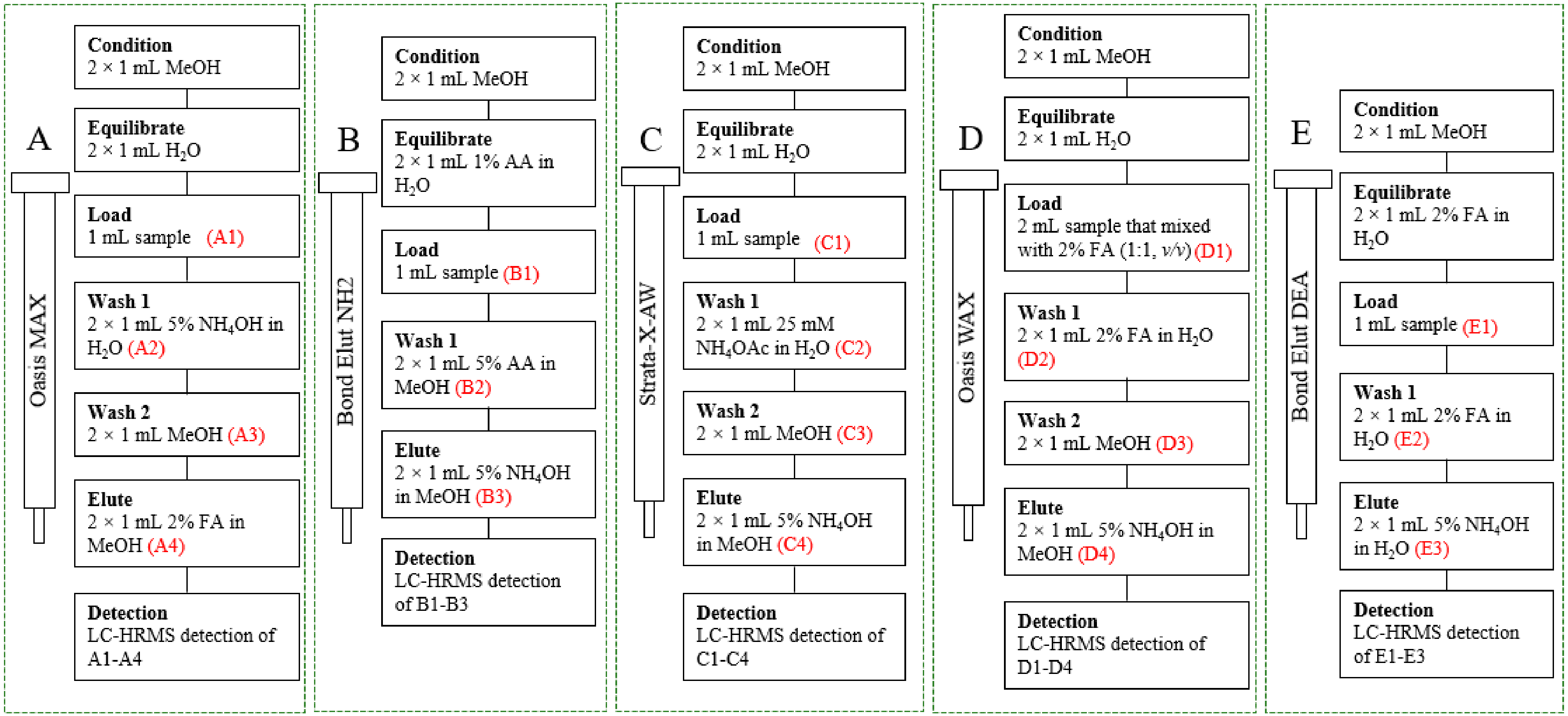

Figure S1. Scheme of purification of glucosinolates extracted from certified rapeseeds (ERM-BC367) with different commercial SPE cartridges: (A) Oasis MAX; (B) Bond Elut NH_2; (C) Strata-X-AW; (D) Oasis WAX; (E) Bond Elut DEA. FA, formic acid; AA, acetic acid; MeOH, methanol; NH_4OH, ammonium hydroxide; NH_4OAc, ammonium acetate.

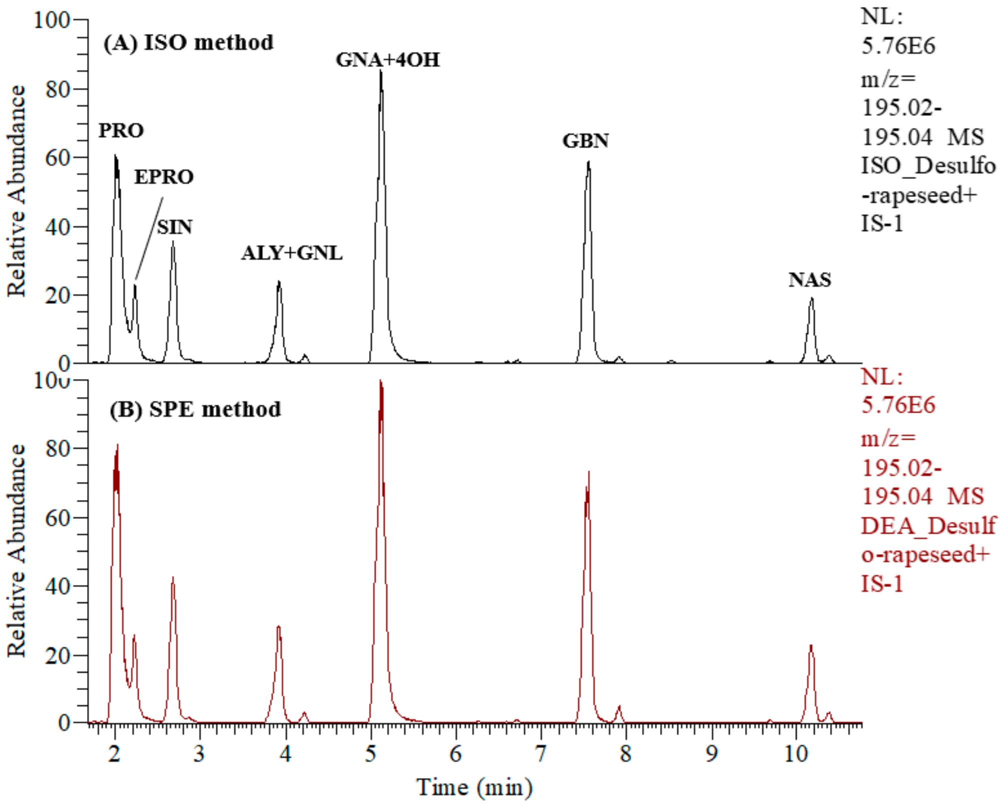

Figure S2. Extracted ion chromatogram (EIC) (m/z 195) of certified rapeseed reference (ERM-BC367) follow (A) the ISO method and (B) SPE method. Progoitrin (PRO); epiprogoitrin (EPRO); glucoalyssin (ALY); gluconapoleiferin (GNL); gluconapin (GNA); 4-hydroxyglucobrassicin (4OH); glucobrassicanapin (GBN); gluconasturtiin (NAS).
